
JOURNAL INFORMATION
==============================
NLM Title Abbreviation: Wirtschaftsdienst
Journal ID: Wirtschaftsdienst
NLM Journal ID: 101086612

Wirtschaftsdienst (Hamburg, Germany : 1949)

ISSN: 0043-6275

ARTICLE INFORMATION
==============================
PMCID: PMC8800411
PMID: 35125548
DOI: 10.1007/s10273-022-3089-4
Article ID: 3089
Article version: 1
Subjects: Zeitgespräch

Not kennt kein Gebot? Sondervermögen als Speicher von Notlagenkrediten

Debt Brake or Breach of the Rules? Building Budget Reserves in Times of Crisis

Büttner Thiess Thiess.Buettner@fau.de

grid.5330.5 0000 0001 2107 3311 LS für Volkswirtschaftslehre, Friedrich-Alexander-Universität Nürnberg-Erlangen, Lange Gasse 20, 90403 Nürnberg, Deutschland
Electronic publication date: 2022 Jan 29
Print publication date: 2022
Volume: 102
Issue: 1
First page: 23
Last page: 26
Copyright: © Der/die Autor:in 2022
License: Open Access: Dieser Artikel wird unter der Creative Commons Namensnennung 4.0 International Lizenz veröffentlicht (https://creativecommons.org/licenses/by/4.0/deed.de).
Open Access wird durch die ZBW — Leibniz-Informationszentrum Wirtschaft gefördert.
License URL: https://creativecommons.org/licenses/by/4.0/

Keywords: E62, H62, H77
issue-copyright-statement: © The Author(s) 2022

==============================
The German federal government is facing criticism for using the exemption from the national debt brake due to the coronavirus crisis to expand the fiscal leeway for its future energy and climate policies. The budget dispute is taking place against the backdrop of a massive expansion of government debt in Europe. In the following, the author explain the critique of the latest supplementary budget and argues that the federal government has missed the opportunity to prove that the fiscal policy options within the existing rules are sufficient.

Prof. Dr. Thiess Büttner ist Inhaber des Lehrstuhls für Volkswirtschaftslehre, insbesondere Finanzwissenschaft, an der Friedrich-Alexander-Universität Erlangen-Nürnberg und Vorsitzender des Beirats des Stabilitätsrats und Mitglied im Wissenschaftlichen Beirat beim Bundesministerium der Finanzen.

Der Umgang der Bundesregierung mit der Schuldenbremse ist von verschiedener Seite scharf kritisiert worden. Bemängelt wird insbesondere, dass die Bundesregierung die Ausnahmeklausel aufgrund der Corona-Krise nutzt, um die künftigen Finanzierungsspielräume für ihre Energie- und Klimapolitik auszuweiten. Der Streit um die Haushaltspolitik vollzieht sich vor dem Hintergrund einer massiven Ausweitung der öffentlichen Verschuldung in Europa. Zugleich werden Rufe nach weiteren europäischen Schulden und Aufweichung der europäischen Fiskalregeln lauter. Die Politik sollte daher alles daran setzen, die Funktionsfähigkeit der Finanzpolitik unter den gegebenen Regeln der Schuldenbremse unter Beweis zu stellen.

## Absicherung der europäischen Fiskalregeln durch die Schuldenbremse

Im Jahr 2009 hat der Bundestag mit der erforderlichen Zwei-Drittel-Mehrheit die Schuldenbremse eingeführt. Die bis dahin geltende Verschuldungsgrenze des Grundgesetzes konnte einen trendmäßigen Anstieg der Verschuldung im Verhältnis zur Wirtschaftsleistung nicht verhindern. Ende der 1990er Jahre hatte Deutschland zudem die eigene Währung aufgegeben und sich verpflichtet, die Stabilitätskriterien einzuhalten. Dies beinhaltet einen Schuldenstand von maximal 60 % der Wirtschaftsleistung und ein Defizit von höchstens 3 %. Im Jahr 2005 wurden die Regeln ergänzt. Defizite im Abschwung müssen nunmehr durch Überschüsse in wirtschaftlich guten Zeiten annähernd ausgeglichen werden. Dies wird durch die Zielvorgabe eines mittelfristigen Haushaltsziels für den von konjunkturellen Schwankungen bereinigten Finanzierungssaldo erreicht. Eine Neuregelung der Verschuldungsgrenze des Grundgesetzes war daher unausweichlich, zumal eine Koordination innerhalb des deutschen Bundesstaats erforderlich war. Mit dem Fiskalvertrag vom März 2012 besteht ohnehin eine Verpflichtung nationale Fiskalregeln festzulegen, die das Einhalten der europäischen Vorgaben sicherstellen. Eine zentrale Vorgabe ist dabei, dass das strukturelle Finanzierungsdefizit einen Wert von 0,5 % der Wirtschaftsleistung nicht überschreiten darf.

Die Schuldenbremse definiert eine Verschuldungsgrenze für den Bund und die Länder. Für den Bund wurde festgelegt, dass das strukturelle Defizit einen Wert von 0,35 % nicht überschreiten darf. Für die Länder gilt eine Obergrenze von 0 %. Der rechnerische Unterschied zum mittelfristigen europäischen Haushaltsziel eröffnet Spielräume für Kommunen, Sozialversicherungen und Extrahaushalte der Länder.

Bei der konkreten Ausgestaltung der Regelungen ist zu beachten, dass die europäischen Fiskalregeln für alle Mitgliedsländer einheitlich auf den Volkswirtschaftlichen Gesamtrechnungen (VGR) aufsetzen. Das sichert die Vergleichbarkeit und vereinfacht eine Überwachung durch die Europäische Kommission. Die konkreten Vorgaben der Schuldenbremse zielen dagegen auf die Haushaltsplanung (Finanzstatistik). Dies erleichtert zwar die Implementierung, allerdings ist es wichtig, sich nicht zu stark von den europäischen Vorgaben zu lösen, um tatsächlich das Einhalten der europäischen Verpflichtungen abzusichern. Insbesondere ist zu beachten, dass nicht nur die Kernhaushalte, sondern auch Extrahaushalte berücksichtigt werden. Dies ist wichtig, da die öffentliche Hand in Deutschland in spürbarem Umfang staatliche Aufgaben über Sondervermögen finanziert, die zum Staatssektor gezählt werden.

## Verbuchung von Sondervermögen

Die konkrete Ausformulierung der Schuldenbremse für den Bund sieht vor, dass neu gegründete Sondervermö-

**Prof. Dr. Thiess Büttner** ist Inhaber des Lehrstuhls für Volkswirtschaftslehre, insbesondere Finanzwissenschaft, an der Friedrich-Alexander-Universität Erlangen-Nürnberg und Vorsitzender des Beirats des Stabilitätsrats und Mitglied im Wissenschaftlichen Beirat beim Bundesministerium der Finanzen.

* Für hilfreiche Anmerkungen und Kommentare zu einer früheren Fassung dankt der Verfasser K. Wendorff. Die Verantwortung für den Text liegt ausschließlich beim Verfasser.

gen bei der Schuldenbremse einbezogen werden. Bislang wurde hierzu die Nettokreditaufnahme mit dem Saldo der Sondervermögen konsolidiert. Demzufolge ist es für die Erfassung in der Schuldenbremse entscheidend, ob Zahlungen aus dem Sondervermögen abfließen. Die Zahlungsströme zwischen dem Kernhaushalt und dem Sondervermögen spielen dagegen keine Rolle. Diese Verbuchung ist gut nachvollziehbar, schafft Transparenz über die Finanzpolitik des Bundes und korrespondiert mit der Verbuchung der öffentlichen Ausgaben im Rahmen der Europäischen Fiskalregeln.

Die neue Bundesregierung hat allerdings beschlossen, die Verbuchungspraxis zu ändern. Künftig soll bei der Berechnung der strukturellen Nettokreditaufnahme im Rahmen der Schuldenbremse nicht mehr der Saldo der Sondervermögen konsolidiert werden. Es soll vielmehr bereits die Übertragung von Haushaltsmitteln vom Kernhaushalt in ein Sondervermögen verbucht werden. Die Bundesregierung verweist in der Begründung auf Schwierigkeiten, Mittelabflüsse zu planen. Diese Begründung überrascht. Da die europäischen Fiskalregeln letztlich auf den Mittelabfluss abstellen, besteht weiterhin die Notwendigkeit, die Mittelabflüsse in der Finanzplanung und der Haushaltskoordinierung zu berücksichtigen, um das Erreichen des mittelfristigen Haushaltsziels zu sichern. Da die Schuldenbremse des Bundes nun nicht mehr alle relevanten Salden erfasst, wird lediglich die Wirksamkeit der Schuldenbremse beeinträchtigt, ein Einhalten der europäischen Fiskalregeln abzusichern.

Der konkrete Anlass der Änderung der Verbuchungsregel ist die Überführung von ungenutzten Kreditermächtigungen im Bundeshaushalt in den Energie- und Klimafonds. Mit der neuen Verbuchungsregel muss der Bund im Jahr der Befüllung der Sondervermögen Verschuldungsspielräume gemäß der Schuldenbremse haben, nicht aber im Jahr des Abflusses. Durch die Änderung können die übertragenen Mittel künftig beliebig zur Finanzierung von Ausgaben verwendet werden, ohne die Einhaltung der Schuldenbremse zu gefährden.[1] Während die neue Verbuchung die erforderlichen Spielräume zunächst nur auf der Zeitachse verschiebt, kommt es im Zusammenspiel mit der Ausnahmeklausel der Schuldenbremse zu einer erheblichen Ausweitung der Verschuldung.

## Außergewöhnliche Notsituation

Im Jahr 2021 lag die Nettokreditaufnahme des Bundes mit einem Wert von rund 240 Mrd. Euro ganz erheblich über dem nach der Schuldenbremse zulässigen Wert. Dieser wird angesichts der Unterauslastung der Wirtschaft von der Bundesregierung mit rund 33 Mrd. Euro angesetzt (Deutscher Bundestag, 2021). Die äußerst umfangreichen Kreditermächtigungen waren nur möglich, weil der Bundestag eine außergewöhnliche Notsituation gemäß Art. 109 Abs. 3 Satz 2 des Grundgesetzes festgestellt hat. Damit ist eine Notlage angesprochen, die sich der Kontrolle des Staates entzieht, und die es erforderlich macht, Maßnahmen zu deren Überwindung und Bewältigung zu treffen, die zur Überschreitung der regulären Grenzen führen. Schon bei dem ursprünglich geplanten Haushalt des Jahres 2021 bestanden Zweifel, dass die bewilligten Finanzmittel für die Bekämpfung der Krise in vollem Umfang benötigt würden (Unabhängiger Beirat des Stabilitätsrats, 2020). Im Vollzug des Haushalts wurden schließlich im Jahr 2021 etwa 180 Mrd. Euro der Kreditermächtigungen zur akuten Bekämpfung der Krise ausgegeben. Die verbleibenden 60 Mrd. Euro wurden mit einem Nachtragshaushalt nun in den Energie- und Klimafonds eingestellt.

Im Jahr 2020 wurden bereits zur Krisenbekämpfung ungenutzte Finanzmittel im Umfang von 28 Mrd. Euro in den Energie- und Klimafonds eingestellt. Schon damals war kritisiert worden, dass die Zweckentfremdung der Mittel nicht von der Notlagenklausel gedeckt ist. Allerdings konnte die damalige Bundesregierung darauf verweisen, dass der künftige Mittelabfluss aus dem Fonds auf den zulässigen Finanzierungsspielraum unter der Schuldenbremse angerechnet würde.[2] Mithin sei eine Umgehung der Schuldenbremse durch die konsequente Verbuchungspraxis zu den Sondervermögen ausgeschlossen. Die von der neuen Bundesregierung beschlossene Änderung der Verbuchungsregeln entfernt nun genau diesen Teil der Schuldenbremse.

Von der Bundesregierung werden die höheren künftigen Ausgaben im Rahmen der Energie- und Klimapolitik mit dem Hinweis auf geringe private Investitionen gerechtfertigt (Deutscher Bundestag, 2021). Tatsächlich haben sich diese Investitionen in der Krise schwächer entwickelt als noch 2019 erwartet wurde. Dies ist aber eine normale Begleiterscheinung eines wirtschaftlichen Einbruchs. Im Aufschwung steigen die Investitionen dann wieder stärker, was auch die Regierungsprognose anzeigt. Gegenüber den Erwartungen des Jahres 2019 werden sich die Verluste am gesamtwirtschaftlichen Kapitalstock daher im weiteren Verlauf in sehr engen Grenzen halten. Es ist sicherlich nicht Intention der Schuldenbremse, die Aus-

1 Zu den Effekten der Änderung der Verbuchung ausführlicher: Unabhängiger Beirat des Stabilitätsrats (2021).

2 So schreibt die Bundesregierung am 17.8.2020 in der Antwort auf eine Anfrage der FDP-Fraktion: „Mit Blick auf die Schuldenregel nach Artikel 115 GG ist zu berücksichtigen, dass eine Anrechnung auf die Kreditobergrenze erst dann erfolgt, wenn die Mittel tatsächlich ausgegeben werden…“ (Deutscher Bundestag, 2020).

nahmeklausel zu nutzen, wenn solche moderaten Abweichungen von Regierungsprognosen entstehen. Es ist daher gut nachvollziehbar, dass in der Übertragung der Mittel an den Energie- und Klimafonds eine verfassungswidrige Zweckentfremdung der Notlagenkredite gesehen wird (hierzu Hennecke, 2021).

Bleibt die Umwidmung der Kreditermächtigungen unbeanstandet, kann die Schuldenbremse im Zusammenwirken mit den neuen Verbuchungsregeln auch in Zukunft bei Vorliegen einer Notsituation unterlaufen werden. Da man sich in Berlin darauf verständigt hat, dass auch für 2022 eine außergewöhnliche Notsituation nach Art. 109 gelten soll, ist es durchaus möglich, dass schon in diesem Jahr weitere kreditfinanzierte Mittel in Sondervermögen nachträglich eingestellt werden.

## Konsequenzen für die Fiskalpolitik

Dass die Zuführung zum Energie- und Klimafonds bei Änderung der Verbuchung eine kreditfinanzierte Ausweitung von Staatsausgaben ermöglicht, wird von Interessengruppen und politischen Parteien begrüßt, die höhere Ausgaben im Rahmen der Energie und Klimapolitik fordern. Manche Volkswirte argumentieren angesichts solcher Forderungen, dass eine zusätzliche Ausweitung der Staatsverschuldung die Tragfähigkeit der Staatsfinanzen derzeit nicht bedroht (Schnitzer und Truger, 2021). Zwar ist dies richtig. Diese Argumentation übersieht aber die weiteren institutionellen Festlegungen im Rahmen der Schuldenbremse. Nach den bestehenden Regelungen können die derzeit aufgenommenen Schulden nicht beliebig prolongiert werden, wie es Makroökonom:innen oft unterstellen. Vielmehr sind alle Kredite wieder zu tilgen, welche die normale Regelgrenze für die Neuverschuldung überschreiten. Jeder zusätzliche durch Verschuldung finanzierte Euro schafft daher eine zukünftige Haushaltslücke im Tilgungszeitraum in gleicher Höhe. Angesichts der bereits feststehenden Verpflichtungen und der demografischen Entwicklung ist der Bundeshaushalt in Zukunft ohnehin unter starkem Konsolidierungsdruck.

Es ist keineswegs so, dass die Einhaltung der bisherigen Regeln der Schuldenbremse erhebliche Konsolidierungsanstrengungen für den Bundeshaushalt erforderte. Der Bundeshaushalt verfügt über erhebliche Rücklagen, die während der Corona-Krise nicht angetastet wurden und deren Verwendung im Rahmen der Schuldenbremse ohne Weiteres möglich ist.

Die Regierung selbst erwartet gemäß ihrer jüngsten Prognose, dass sich die Wirtschaft bereits 2022 wieder in einer konjunkturellen Normallage befindet. Wenn die Fiskalpolitik nun auch nach der von der Regierung prognostizierten Rückkehr zur Normalauslastung expansiver sein soll, indem zusätzliche Haushaltsspielräume geschaffen werden, agiert sie nicht etwa anti-, sondern prozyklisch.

## Europäische Perspektive

Unter den Regeln der Schuldenbremse konnte die deutsche Finanzpolitik 2020 und 2021 einen wichtigen Beitrag zur Stabilisierung leisten. Die Ausrichtung am Auslastungsgrad ermöglichte eine deutlich erhöhte Regelverschuldung. Die Notlagenklausel stellte darüber hinaus die Finanzierung der erforderlichen Maßnahmen zur Bekämpfung und Überwindung der Notsituation sicher. Da in den anderen EU Ländern entsprechende nationale Fiskalregeln gelten und auf EU Ebene die generelle Ausnahme von den Fiskalregeln beschlossen wurde, war eine adäquate finanzpolitische Reaktion in allen Mitgliedsländern möglich. Zugleich ist die Verschuldung aber massiv gestiegen. Die Europäische Kommission schätzt, dass der staatliche Schuldenstand in der Eurozone sich mittlerweile auf einen Wert von 100 % der Wertschöpfung beläuft.

Europaweit ist die Wirtschaft deutlich auf Erholungskurs. So prognostiziert die Europäische Kommission für die Eurozone eine Rückkehr zur Normalauslastung im Jahr 2022. Nach dem unerwartet starken Preisauftrieb 2021 räumt die EZB inzwischen ein, dass die Inflationsrate auch 2022 deutlich oberhalb ihres Zielwertes liegen wird.[3] Dies weckt Erwartungen an eine geldpolitische Reaktion zur Sicherung der Geldwertstabilität. Die wachsende Verschuldung in der Eurozone führt aber zunehmend in eine Situation der fiskalischen Dominanz, in der eine solche Reaktion vor dem Hintergrund der Effekte auf die öffentlichen Haushalte herausgezögert wird oder ganz unterbleibt. Das würde die Geldwertstabilität des Euro nachhaltig beeinträchtigen.

Für die Finanzpolitik in Europa ist es daher an der Zeit, zum mittelfristigen Haushaltsziel zurückzukehren. Nach den europäischen Fiskalregeln ist dabei nur eine graduelle Verringerung der strukturellen Salden um jährlich 0,5 % der Wirtschaftsleistung erforderlich. Dies kann nach derzeitiger Sicht in allen Mitgliedsländern weitgehend ohne zusätzliche Konsolidierungsmaßnahmen geschehen, da schon die Rücknahme der Corona-Maßnahmen die strukturellen Finanzierungssalden verbessert (Unabhängiger Beirat des Stabilitätsrats, 2021).

Die EU-Staaten haben sich nicht zuletzt auf Betreiben Deutschlands im Fiskalvertrag dazu verpflichtet, nationale Fiskalregeln einzurichten, die nach dem Muster der

3 Die jüngste Prognose beinhaltet eine Inflationsrate von 3,2 % (harmonisierter Verbraucherpreisindex) (EZB, 2021).

Schuldenbremse die Neuverschuldung am mittelfristigen Haushaltsziel ausrichten. Letztlich ging es darum, in ganz Europa die Haushaltsdisziplin zu verbessern und gesunde öffentliche Finanzen der Mitgliedstaaten zu erreichen. Dies sollte auch heute weiter ein Kernanliegen der deutschen Finanzpolitik sein, nicht zuletzt, weil die fiskalischen Risiken der europäischen Finanzpolitik gerade für Deutschland weiter zugenommen haben, man denke nur an die erheblichen Garantien für die neue Verschuldung der EU (Heinemann, 2021).

Angesichts der fortgesetzten Bestrebungen in weiten Teilen Europas, die Fiskalregeln weiter zu verwässern, und der Forderungen nach weiteren europäischen Schulden, wäre die neue Bundesregierung im Interesse der Stabilität des Euro gut beraten, die Funktionsfähigkeit der Finanzpolitik unter den gegebenen Regeln der Schuldenbremse unter Beweis zu stellen. Die Chance hierzu hat die Bundesregierung nun vertan. Es steht zu befürchten, dass dies die deutsche Position in den Verhandlungen um die europäische Finanzpolitik in einem kritischen Moment weiter schwächt.



Literatur

Deutscher Bundestag (2020), Drucksache 19/21638.
Deutscher Bundestag (2021), Entwurf eines Gesetzes über die Feststellung eines Zweiten Nachtrags zum Bundeshaushaltsplan für das Haushaltsjahr 2021, Drucksache 20/300.
EZB (2021), Eurosystem staff macroeconomic projections for the euro area, 16. Dezember.
Heinemann F Die Überdeckung der Next Generation EU-Schulden im neuen EU-Eigenmittelbeschluss: Ausmaß und Haftungskonsequenzen List Forum 2021 47 133 150 10.1007/s41025-021-00234-3
Henneke, H.-G. (2021), Stellungnahme zur Anhörung des Haushaltsausschusses des Deutschen Bundestages am 10.1.2022 zum Entwurf eines 2. Nachtragshaushaltsgesetzes 2021 (BT-Drs. 20/300).
Schnitzer, M. und A. Truger, (2021), Etwas mehr Pragmatismus, bitte! Investitionen auch über Kredite zu finanzieren, führt nicht in die Schuldenkrise, Frankfurter Allgemeine Zeitung, vom 28. November.
Unabhängiger Beirat des Stabilitätsrats (2020), 5. Stellungnahme des unabhängigen Beirats des Stabilitätsrats vom 14. Dezember 2020.
Unabhängiger Beirat des Stabilitätsrats (2021), 17. Stellungnahme des unabhängigen Beirats des Stabilitätsrats vom 7. Dezember 2021.
